# Intranasal Infection with *Chlamydia abortus* Induces Dose-Dependent Latency and Abortion in Sheep

**DOI:** 10.1371/journal.pone.0057950

**Published:** 2013-02-28

**Authors:** David Longbottom, Morag Livingstone, Stephen Maley, Arjan van der Zon, Mara Rocchi, Kim Wilson, Nicholas Wheelhouse, Mark Dagleish, Kevin Aitchison, Sean Wattegedera, Mintu Nath, Gary Entrican, David Buxton

**Affiliations:** 1 Moredun Research Institute, Edinburgh, United Kingdom; 2 Faculty of Veterinary Medicine, University of Utrecht, Utrecht, The Netherlands; 3 Biomathematics and Statistics Scotland, Edinburgh, United Kingdom; University of California San Francisco, University of California, Berkeley, and the Children's Hospital Oakland Research Institute, United States of America

## Abstract

**Background:**

Latency is a key feature of the animal pathogen *Chlamydia abortus*, where infection remains inapparent in the non-pregnant animal and only becomes evident during a subsequent pregnancy. Often the first sign that an animal is infected is abortion occurring late in gestation. Despite this, little is understood of the underlying mechanisms that control latency or the recrudescence of infection that occurs during subsequent pregnancy. The aim of this study was to develop an experimental model of latency by mimicking the natural route of infection through the intranasal inoculation of non-pregnant sheep with *C. abortus*.

**Methodology/Principal Findings:**

Three groups of sheep (groups 1, 2 and 3) were experimentally infected with different doses of *C. abortus* (5×10^3^, 5×10^5^ and 5×10^7^ inclusion forming units (IFU), respectively) prior to mating and monitored over 2 breeding cycles for clinical, microbiological, pathological, immunological and serological outcomes. Two further groups received either negative control inoculum (group 4a,b) or were inoculated subcutaneously on day 70 of gestation with 2×10^6^ IFU *C. abortus* (group 5). Animals in groups 1, 2 and 5 experienced an abortion rate of 50–67%, while only one animal aborted in group 3 and none in group 4a,b. Pathological, microbiological, immunological and serological analyses support the view that the maternal protective immune response is influenced by initial exposure to the bacterium.

**Conclusions/Significance:**

The results show that intranasal administration of non-pregnant sheep with a low/medium dose of *C. abortus* results in a latent infection that leads in a subsequent pregnancy to infection of the placenta and abortion. In contrast a high dose stimulates protective immunity, resulting in a much lower abortion rate. This model will be useful in understanding the mechanisms of infection underlying latency and onset of disease, as well as in the development of novel therapeutics and vaccines for controlling infection.

## Introduction

The obligate, intracellular, Gram-negative bacterium *Chlamydia abortus* (*C. abortus*) is recognised as a major cause of abortion and lamb loss throughout the world, with infection resulting in the disease known as Enzootic Abortion of Ewes (EAE) or Ovine Enzootic Abortion (OEA) [1]. Typically, following infection, a vaginal discharge containing large numbers of chlamydial elementary bodies (EBs) may be observed 24–48 hours before abortion, which occurs in the last two to three weeks of normal gestation, although in some cases it may occur earlier [2], [3]. Early research shows that when a ewe or a ewe lamb is exposed to natural infection, there is no apparent immediate clinical effect with the infection remaining latent until the animal becomes pregnant, after which the organism invades the placenta and multiplies, eventually causing abortion [4].

Studies on the pathogenesis of infection have shown that lesions are mostly confined to the placental membranes [5], [6], although they may also occur in the brain and liver of foetuses [7]. Placental lesions initially develop in the hilus of the placentome and extend out to involve the intercotyledonary membranes as well as deeper elements of the placentome [5], [8]–[11]. The fact that typical abortions are seen in the last few weeks of pregnancy [2], [3] correlates with the finding that experimentally-induced placental lesions are initiated at the same time and develop progressively from 110 days of gestation (dg) [5], [9].

While latency of infection and its subsequent recrudescence to trigger the onset of placental changes at a given time are recognised as characteristic features of EAE, the underlying mechanisms that control this series of events are poorly understood. Reproduction of latent infection and subsequent abortion has been previously demonstrated in a small number of ewes that were exposed to *C. abortus* infection in the previous lambing season [12], as well as in ewes parenterally inoculated (subcutaneously, intravenously and intradermally) prior to mating [6]. Furthermore, oral administration of pregnant ewes with chlamydial organisms has been shown to induce a placental infection [6], [12], [13], as has targeted administration of the bacterium over the tonsils [14]. Most recently, Gutierrez et al. [15] have induced placental infection following the oral administration of a high dose of *C. abortus* (5×10^9^ infection-forming units (IFU)) prior to pregnancy, thus establishing latency. Observations therefore implicate the oro-nasal route as the natural port of entry for *C. abortus* in EAE and also indicate that latency may play an important, if not crucial, part in the pathogenesis of infection. The presence of mucosal lymphoid tissue in the ovine nasopharyngeal tract has been observed by Stanley and colleagues [16] and it has been further demonstrated that this nasal-associated lymphoid tissue (NALT) had the characteristics of other mucosal-associated lymphoid tissues and could be an effective mode of delivery for specific pathogens, to mimic the natural acquisition of infection [17].

The aim of this study was therefore to experimentally reproduce latency in non-pregnant sheep following intranasal inoculation with *C. abortus* in order to mimic the natural route of infection. In addition, the study aimed to establish the optimum dose of organisms required for eliciting latency. It was postulated that subsequent pregnancy would then result in recrudescence of infection, which would initiate placental infection and ultimately lead to late-term abortions. The clinical, microbiological, pathological, immunological and serological outcomes and changes were monitored over the course of two lambing seasons.

## Materials and Methods

### Ethics Statement

This study was carried out in strict accordance with the Animals (Scientific Procedures) Act 1986 and in compliance with all UK Home Office Inspectorate regulations. The experimental protocol was approved by the Moredun Experiments and Ethical Review Committee (Permit number: E37/07). All animals were monitored throughout the two year study for any clinical signs at least three times daily and all findings recorded. Any animal found to be suffering or requiring treatment were given appropriate veterinary care in accordance with standard veterinary practice.

### Preparation of Inoculum

*Chlamydia abortus* strain S26/3, isolated at Moredun Research Institute in Scotland in 1979 from a vaccinated ewe that aborted [18], was grown in fertile hens’ eggs and inoculum prepared from infected yolk sacs as previously described [19]. Briefly, infected yolk sacs were ground up using a mortar and pestle with sterile sand, suspended in phosphate-buffered saline (PBS) and centrifuged at 500×g for 10 min to remove gross debris. The middle layer was carefully removed, aliquoted and stored in liquid nitrogen. The titre was determined by inoculating 10-fold dilutions of the stored yolk sac material onto McCoy cell monolayers on coverslips as previously described [20]. Control inoculum was prepared in the same way from uninfected yolk sac material. The inoculum was diluted in PBS to provide the appropriate challenge dose for each group.

### Intranasal Inoculation Over Nasal-associated Lymphoid Tissue

To administer the inoculum evenly over the pharyngeal mucous membrane a disposable plastic syringe was connected via a luer fitting to a plastic tube of 11 cm in length and with an outside diameter of 6 mm. The distal end of the tube was fitted with a smooth solid plastic cap 8 mm in diameter perforated in the centre with a hole 0.5 mm in diameter and 5 mm in depth. Preliminary trials, using 2 ml of coloured fluid and an ovine cadaver, showed that if the tube was fully inserted into one of the nostrils and the fluid was expelled with a steady even pressure, a fine cone-shaped spray was produced that evenly coated the mucous membrane investing the pharyngeal cavity. For the actual inoculation of each ewe, 2 ml of a given inoculum was drawn up into a 5 ml syringe that was then capped and placed in a rack in a chilled container and the animals inoculated without delay as detailed above.

### Study Design

Seventy-nine Scottish Blackface sheep (aged 3 to 5 years) were pre-screened by rOMP90-3 enzyme-linked immunosorbent assay (ELISA) [21] to ensure they were seronegative for *C. abortus*. The sheep were also pre-screened for cellular responses to *C.abortus* antigens by stimulating peripheral blood mononuclear cells (PBMC) with UV-killed *C. abortus* antigen or with the T cell mitogen Con A as a positive control (extract from *Concanavalia ensiformis*, ICN Biochemicals, Cleveland, OH, USA) and the culture supernatants analysed for the presence of interferon-gamma (IFN-γ) (see **Interferon-gamma** **analysis** for full description of methods). We have previously observed significant variation in mitogen-driven IFN-γ production between outbred sheep [22]. Therefore, to avoid an unbalanced effect of cellular responses between experimental groups, the sheep were ranked into low, medium and high ConA responders and subsequently randomly allocated across five groups (data not shown). Groups 1 to 3 each contained 21 animals, while groups 4a and 5 each comprised 8 ewes. Those in group 1 were each inoculated intranasally (i/n) with 5×10^3^ IFU of *C. abortus*, those in group 2 each received 5×10^5^ IFU and those in group 3 were each given 5×10^7^ IFU. Animals in group 4a were each given control inoculum (uninfected yolk sac) i/n. Rectal temperatures were measured and recorded daily for each ewe in groups 1 to 4 from the day of i/n inoculation for a total of 27 days. Rectal temperature data was expressed as the number of animals with a temperature of 40°C or above on a given day. The 8 ewes assigned to group 5 were left untreated at this stage.

Eight weeks after i/n inoculation all ewes were synchronised with progesterone sponges (Veramix, Upjohn Ltd, Crawley, UK) and mated. A total of 16, 18, 19, 8 and 6 ewes became pregnant, in groups 1 to 5 respectively. At 70 dg the 6 pregnant ewes in group 5 were each inoculated subcutaneously (s/c) over the left prefemoral lymph node with 2 ml of inoculum containing 2×10^6^ IFU of *C. abortus*. These animals, which served as the standard challenge control group [23], had their rectal temperatures recorded daily from the day of inoculation. Groups 1 to 3 were housed in three separate pens within one building and were fed on a normal maintenance diet with free access to hay and water. Groups 4a and 5 were housed separately and in different buildings to avoid possible cross-contamination between the groups and were fed and watered, as previously described.

In the following year 16 ewes from group 1, 17 from group 2 and 17 from group 3 were available. A further 10 animals were screened and selected as negative controls (group 4b). All were synchronised, mated and allowed to go through to lamb, as described above. The viability of lambs was monitored and samples collected for pathological, microbiological and serological examination for both year 1 and 2.

### Sample Collection

In the event of an abortion, stillbirth, neonatal mortality or the birth of a live clinically healthy lamb, the placenta was collected and examined for macroscopic lesions typical of EAE [1]. Where possible, affected placental cotyledons were excised, using aseptic techniques, placed in 4 ml sucrose-phosphate-glutamate buffer (SPG) [24] and stored frozen at −20°C for subsequent analysis by quantitative real-time PCR (qPCR) (see below). Similar samples were also taken and stored at 4°C for the subsequent preparation of smears for staining by the modified Ziehl-Neelsen (mZN) method [3]. Smears were examined under high-power microscopy for the presence of chlamydial elementary bodies (EBs) and any other contaminating bacteria. Where the placenta appeared uninfected, cotyledons were excised from 3 different areas and stored and analysed as for infected tissue. At least 2 cotyledons from each placenta collected, including surrounding placental membranes, were placed in 10% formol saline (FS) for histopathological examination. For each foetus recovered, samples of brain, lung, heart and liver were placed in 10% FS for histopathological examination. In addition to placenta, vaginal swabs were collected from all ewes at parturition and stored. They were subsequently analysed by qPCR and mZN, as for cotyledons.

Serum samples were collected prior to inoculation and at regular intervals throughout the study for analysis by the rOMP90-3 ELISA [21] and also for immunological analysis. All foetuses and lambs were sexed and weighed. Lambs that were born live but were weak were given full supportive care, including maternal or commercial colostrum by stomach tube or bottle and supplementary heat. No lambs were reared artificially for more than the first 2 days of life. Any lambs that died subsequently were subject to a post mortem examination and samples collected as above. In the event of an abortion before day 126 of pregnancy, 2 vaginal swabs were taken and used to set up cultures by standard bacteriological methods to detect other bacterial pathogens, such as *Campylobacter* spp and *Salmonella* spp.

### Quantitative Real-time PCR

As placentas were not available for all ewes and because the sampling of a small diseased area is not necessarily reflective of the disease state of the total tissue, vaginal swabs were used to estimate bacterial load. Two vaginal swabs were vigorously vortexed in 1 ml PBS and all liquid removed following centrifugation at 14,000 rpm for 10 min. DNA was extracted from the pellet using a DNeasy® Blood and Tissue Kit (Qiagen Ltd., Crawley, UK) and analysed using the *C. abortus* MOMP-based quantitative real-time PCR (qPCR) protocol described by Livingstone et al. [20]. Results were expressed as the number of *C. abortus* genome copies per µl swab extract total gDNA, as described previously [20].

### Histopathological Examination

Placental samples were fixed for 4–10 days in 10% FS and then trimmed and processed through graded alcohols and a xylene step and embedded in paraffin wax, by standard protocols. Serial sections 5 µm thick were cut, stained with haematoxylin and eosin (H&E) and examined under a microscope for the presence of pathological changes.

In addition, blocks of lung, heart, liver and brain from aborted foetuses and dead lambs were also prepared. From the brain, coronal slices through the frontal cerebrum and striatum (at the level of the caudate nucleus), parietal cerebrum and thalamus (at the level of the optic chiasma), occipital cerebrum and hippocampus, midbrain (at the level of the rostral colliculi), the cerebellar peduncles, the medulla (at the level of the obex) and a sagittal section of cerebellum were taken for detailed histopathological examination.

### Immunohistochemical Analysis

Duplicate sections of the placental samples prepared for H&E examination were incubated with a mouse monoclonal antibody (mAb) to the lipopolysaccharide (LPS) of *C. abortus* strain S26/3 (mAb 13/5) [25]. Bound antibody was detected using the Vectastain Elite ABC kit (Vector Laboratories, Peterborough, UK) with 0.05% 3,3′-diaminobenzidine tetrahydrochloride (DAB) in 0.1 M Tris-HCl, pH7.2/0.01% hydrogen peroxide as substrate. Sections were washed in water, counterstained with haematoxylin, processed through graded alcohols and a xylene step and mounted under coverslips. Negative control sections using an irrelevant monoclonal antibody and positive control sections taken from archived cases of ovine chlamydial abortion were treated similarly.

### Serological Analysis

Serum samples, prepared from blood collected throughout the study, were analysed by rOMP90B-3 ELISA, as previously described [21]. Optical densities were normalized using positive and negative control sera and then expressed as a percentage of the positive control using the following formula:{(OD sample – OD negative control)/(OD positive control – OD negative control)} ×100. Sera with values greater than 60% were considered positive [21].

### Interferon-gamma Analysis

PBMC were isolated from venous blood collected into vacutainer tubes (20 ml/sheep) containing heparin (Becton Dickinson, Cambridge, UK) and cultured for 96 hours at a density of 2×10^6^/ml, according to previously-described protocols [26]. PBMC were stimulated with UV-killed *C. abortus* antigen prepared from a lysate of infected Hep2 cells that contains a mixture of bacterial elementary bodies and reticulate bodies. The lysate was titrated for optimal IFN-γ production by PBMC from post-abortion immune sheep and the same preparation used at a final concentration of 1∶4,500 throughout the experiment. Antigen-specific recall responses were assessed by analysis of the culture supernatants collected after 96 hours for the presence of IFN-γ, the strongest known correlate of infection and immunological protection against EAE [27]. Supernatants were stored at −70°C until further analysis. IFN-γ was measured using the BOVIGAM™ commercial ELISA that cross-reacts with ovine IFN-γ (Prionics, Schlieren-Zurich, Switzerland), according to manufacturer's instructions. Quantification was performed by generating a standard curve using known concentrations of recombinant bovine IFN-γ, as previously described [28].

### Statistical Analysis

Elevated rectal temperature events (temperatures >40°C) were recorded on the first day of occurrence. Data measuring time to such events were analysed using a parametric survival model and assuming an exponential distribution of the data. Days 0 and 1 post-infection were disregarded, as temperature spikes were assumed to occur as a result of the handling procedures. The fitted model included ‘group’ (groups 1, 2 and 3) as the explanatory variable. Animals with normal temperatures (i.e. animals that did not have elevated temperatures) during the experimental period were treated as censored data. Outputs from this parametric survival regression model (probabilities of a normal temperature event and associated 95% confidence intervals at different time points) were then used to identify the periods when the mean occurrence of temperature events differed between the groups, and a separate 3×2 (3 groups and 2 temperature categories) contingency table for the occurrence of temperature events was obtained for each period. The difference in mean frequencies between groups and temperature categories in each period were tested using a Fisher’s exact test. During each identified period, the total number of animals with elevated or normal temperatures, as well as the total number of occurrences of elevated and normal temperature events in each group, was obtained (Supplementary Figure S1).

Data on the incidence of abortion was modelled using a generalised linear model, assuming a Bernoulli distribution for the data. The model included ‘group’ as an explanatory variable and the overall statistical significance of the group effect was assessed using a chi-square statistic. PCR quantification data were transformed on a logarithmic scale (base 10) and a linear model was fitted, including ‘group’ and ‘lambing status’ as well as their interaction as fixed effects. The model also considered heterogeneity in variance due to lambing status. The estimates of variance were obtained using the restricted maximum likelihood (REML) method and an overall statistical significance of a fixed effect was evaluated using the conditional *F*-statistic. Serological (ELISA) and IFN-γ data at different time points were normalised using a logarithmic transformation (base exponential) and analysed with a linear mixed model, using a power model to specify the temporal covariance structure. The linear mixed model included the animal as a random effect; and the ‘group’, ‘time’ (in weeks) and ‘lambing status’, as well as the different 2 and 3-way interaction effects, as fixed effects. The model also included gestational length (as a deviation from the overall mean) as a potential covariate. Parameters of the linear mixed models were estimated using the REML method and statistical significances of fixed effects or their interaction effects were assessed using the modified *F*-statistic.

For all response variables, if multiple comparisons of groups were conducted, then the 2-sided probabilities for each group comparison were obtained; these probabilities were then adjusted using a False Discovery Rate approach (FDR) [29]. The FDR adjusted *P* value (denoted in this paper by *P_f_*) represents the minimum FDR for which the observed difference and associated *P* value would be accepted as statistically significant. All statistical analyses were carried out using the R software version 2.13.1 [30] except the repeated measures analysis of serological and IFN-γ data, which were conducted in GenStat version 14.1 software (VSN International, Hemel Hempstead, UK).

## Results

### Clinical Outcome of Inoculation with *C. abortus* (Year 1)

**Figure 1:**
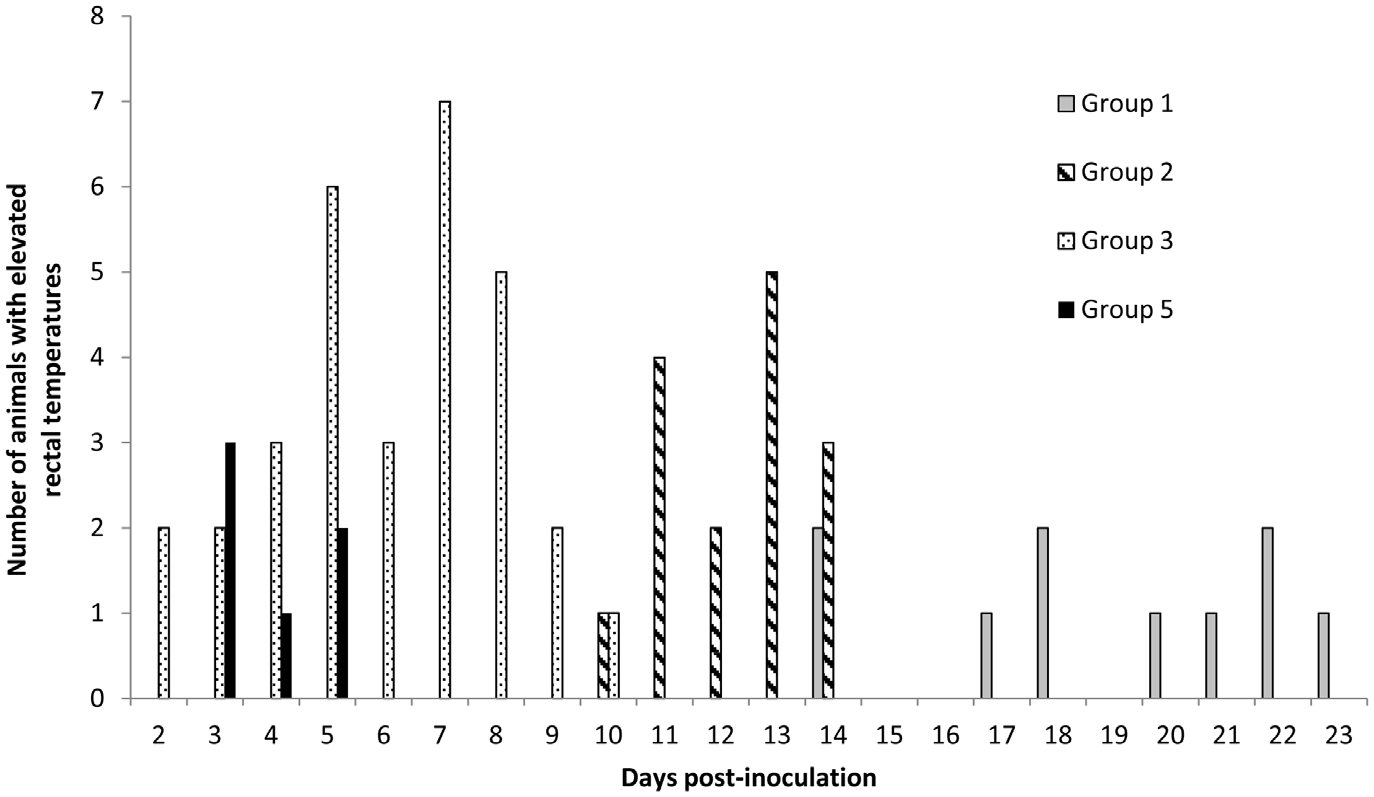
Number of ewes recorded daily with elevated (≥40°C) rectal temperatures following intranasal inoculation with 5 ×**10^3^ (Group 1), 5**×**10^5^ (Group 2) or 5**×**10^7^ (Group 3) IFU** ***C. abortus*** **, or subcutaneously injected with 2**×**10^6^ IFU at 70 days of gestation (Group 5).** None of the ewes inoculated with control inoculum (Group 4a) developed elevated rectal temperatures.

Groups 1–4 were inoculated prior to mating and group 5 at 70 days of gestation (dg) and the rectal temperature readings recorded (Figure 1). Only ewes that developed an elevated temperature (≥40°C) from day 2 post inoculation (dpi) onwards were considered for further analysis, as temperature events prior to this time were considered to have been influenced by handling and so were disregarded. A total of 16 of the starting 21 non-pregnant ewes from group 3 were recorded as having developed a fever, which occurred from day 2 post inoculation (dpi) until day 10. The group 2 animals developed a raised temperature from 10–14 dpi that affected 6 of the non-pregnant ewes and 6 of the ewes in group 3 had short-lived febrile episodes from 14–23 dpi. No febrile responses were recorded for the 8 non-pregnant ewes in group 4a. In group 5, 5 of the 6 pregnant ewes were recorded as febrile from 3 to 5 dpi.

Based on the survival regression model, the risk of animals in group 3 developing a raised temperature was statistically significantly higher than for group 1 (*P_f_* <0.001) and group 2 (*P_f_* <0.001). The mean hazard ratios of developing a raised temperature in group 3 were estimated as 6.11 (95% CI: 2.39, 15.63) and 5.67 (95% CI: 2.22, 14.49) times higher relative to group 1 and group 2 respectively. However, there was no evidence that the mean hazard ratio of group 1 relative to group 2 (0.93; 95% CI: 0.30, 2.87) was statistically significant (*P_f_*  = 0.896).

A survival curve demonstrating the mean proportion of animals that exhibited a normal temperature in each treatment group at different time points (at 24h intervals) is presented in Supplementary Figure S1. A clear pattern emerged regarding the risk of developing an elevated temperature for animals of different groups at different days post inoculation. It was evident that the mean risk in group 3 was higher until 8 dpi, with the mean proportion of animals not exhibiting an elevated temperature by 8 dpi being only 0.24 (95% lower and upper confidence intervals (CI): 0.11, 0.51). Animals in group 2 were at a higher risk during the period of 10 to 13 dpi, with the mean proportion of animals exhibiting a normal temperature by 13 dpi being 0.71 (95% CI: 0.55, 0.94). Animals in group 1 were at a higher risk during the period of 14 to 22 dpi and the estimate of the mean proportion of animals with normal temperature by 22 dpi was 0.71 (95% CI: 0.55, 0.94). This analysis enabled the data to be categorised into 3 distinct periods according to the manifestation of an elevated temperature in the different groups: period 1 (3 to 8 dpi), period 2 (9 to 13 dpi) and period 3 (14 to 26 dpi). The Fisher’s exact test showed that there was strong evidence (*P_f_* <0.01) of an association between the mean proportion of ewes with elevated temperatures and the 3 groups at each period. It has been noted that proportionately a greater number of ewes in groups 3, 2 and 1 exhibited an elevated temperature during the periods 1, 2 and 3, respectively. Similar observations were made for the mean total number of occurrences of elevated temperature events with respect to group category at each period (*P_f_* <0.01).

### Clinical Outcome of Pregnancy

For the purposes of calculation, ewes were deemed to have aborted if they produced one or more dead foetuses or gave birth to live non-viable lambs that died within 48 hrs (i.e. neonatal deaths and stillbirths). Abortions were judged to be due to *C. abortus* if antigen could be demonstrated unequivocally by mZN and either qPCR or by pathological findings.

The outcomes of pregnancy for each of the 5 groups are presented in Table 1. Of the 16 pregnant ewes in group 1, 10 aborted, producing a total of 8 dead foetuses and 4 non-viable lambs. Gestation of aborted ewes ranged from 83 to 137 days, with 2 ewes aborting before 100 dg. In group 2, 12 of the 18 ewes aborted, producing 9 dead foetuses and 8 non-viable lambs. Gestation for group 2 aborted ewes ranged from 74 to 138 days, again with 2 ewes aborting before 100 dg. In group 3, one of 19 pregnant ewes aborted a single foetus at 115 dg.

**Table 1:** The clinical outcome of pregnancy in year 1 for ewes intranasally inoculated with *C. abortus* prior to pregnancy (group1–3), intranasally administered control inoculum (group 4a) or subcutaneously injected with *C. abortus* at 70 days of gestation (group 5).

Group	Number of ewes				Number of lambs		
	Pregnant	Lambed (%)	Aborted (%)	Mean gestational length	Viable	Non-viable^a^	Dead
1	16	6 (41)	10 (59)	125	8	4	8
2	18	6 (33)	12 (67)	127	7	8	9
3	19	18 (95)	1 (5)	141	25	1^b^	1
4a	8	8 (100)	0 (0)	144	10	1^b^	0
5	6	3 (50)	3 (50)	134	4	0	5

a Includes neonatal deaths (born live but died within 48 hrs) and stillbirths.

b Death due to dystocia.

There were no abortions in any of the negative control group 4a animals, whereas 3 of the 6 ewes in positive control group 5 aborted a total of 5 foetuses (range 119 to 127 dg). The remaining 6 ewes in group 1 produced 8 live viable lambs between 140 and 146dg. Six group 2 ewes produced 7 live lambs (136 to147 dg), while in group 3, 18 ewes gave birth to a total of 25 live lambs (135 to 148 dg), including a ewe that had a live lamb and a sibling that died as a result of dystocia. In group 4a all ewes had live lambs (n = 10), although one ewe also had a lamb that died due to dystocia. In group 5, 3 ewes had 4 live viable lambs (141–146 dg).

There was no statistical evidence (*P_f_* = 0.800) of a difference in the mean proportion of abortion cases between group 1 (0.63, 95% CI: 0.38, 0.82) and 2 (0.67, 95% CI: 0.43, 0.84) ewes. However, the mean proportion of abortion cases in ewes of group 3 (0.05, 95% CI: 0.01, 0.29) was statistically significantly (*P_f_* = 0.005) smaller compared to groups 1 and 2.

### Detection of *C. abortus*

The results of the detection of *C. abortus* in placental tissues by mZN and on vaginal swabs by qPCR are presented in Table 2. Of the ewes that aborted in groups 1–3 and 5, evidence of EAE, typified by macroscopic gross lesions, was observed in the placentas. Large numbers of *C. abortus* organisms were demonstrated in all abortion placental samples from all groups by mZN. Presence of the pathogen was confirmed on vaginal swabs taken from aborted ewes and organism load quantified by qPCR. The mean number of organisms detected in aborted ewes from groups 1, 2 and 5 was very similar (4.2×10^6^, 4.7×10^6^ and 4.8×10^6^ genomes, respectively), whereas the mean number of organisms detected in group 3 was lower (2.4×10^5^) but was represented by only one aborted ewe.

**Table 2:** Detection of pathological changes, *C. abortus* organisms and genomic DNA in the placentas of ewes intranasally inoculated with *C. abortus* prior to pregnancy (group1–3), intranasally administered control inoculum (group 4a) or subcutaneously injected with *C. abortus* at 70 days of gestation (group 5).

Group	Pregnancy outcome^a^	Lesions^b^	mZN^c^	qPCR^d^
1	LambedAborted	2+, 4−7+, 3+/−	2+, 4−10+	7.43×10^4^ (6.15×10^4^) [1.13×10^3^]4.20×10^6^ (1.09×10^6^) [3.54×10^6^]
2	LambedAborted	1+/−, 5−7+, 2+/−, 1−, 2 ns	2+, 4−12+	8.52×10^2^ (7.50×10^2^) [1.22×10^2^]4.73×10^6^ (1.03×10^6^) [4.43×10^6^]
3	LambedAborted	3+, 15−e1+/−	3+, 15−e1+	4.34×10^5^ (2.84×10^5^) [6.85×10^1^]2.36×10^5^
4a	LambedAborted	8−eN/A	8−eN/A	2.4×10^1^ (1.4×10^1^) [1.1×10^1^]^f^NA
5	LambedAborted	1+, 2−3+	1+, 2−3+	7.15×10^2^ (5.49×10^2^) [1.93×10^2^]4.76×10^6^ (1.69×10^6^) [5.41×10^6^]

a see Table 1.

b number of ewes with histopathological lesions in placental samples: +, lesions characteristic of *C. abortus* infection; +/−, mild non-specific changes; −, no lesions observed; ns, no samples available.

c detection of chlamydial EBs following mZN staining of smears (swabs used where placentas were not available): +, positive for chlamydial antigen; −, no EBs detected.

d mean (+/− SEM) [median] of the number of *C. abortus* genomes detected per µl total DNA swab extract.

e includes lamb that died as a result of dystocia.

f considered negative as below the background level of 40 genome copies.

*C. abortus* was also evident in cases where ewes produced clinically healthy lambs. Two ewes in each of groups 1 and 2, 3 ewes in group 3 and one ewe in group 5 were positive by mZN and also showed a variable bacterial load when tested by qPCR (7.1×10^2^–4.3×10^5^ genomes). The reason for the variability appeared to be a consequence of individual animals in each of groups 1 and 3 skewing the mean of the data, as demonstrated by the difference between mean and median results in Table 2. The remaining ewes, including those in group 4a that gave birth to live viable offspring, were negative by mZN and only low numbers of genomes were detected by qPCR (Group 4a animals were considered negative as samples were below the limit of detection for the qPCR (background level of approx. 40 genome copies)).

Irrespective of whether an animal aborted or lambed, there was no statistically significant difference (*P* = 0.26) in mean bacterial loads between animals in groups 1, 2 and 3. Irrespective of dose or route of infection, the mean bacterial load from ewes that lambed (in log10 scale, 2.39; 95% CI: 1.81, 2.97) was statistically significantly (*P*<0.001) lower in comparison to the mean bacterial load from ewes that aborted (in log10 scale, 6.35; 95% CI: 6.10, 6.60).

### Histopathology and Antigen Detection

**Figure 2:**
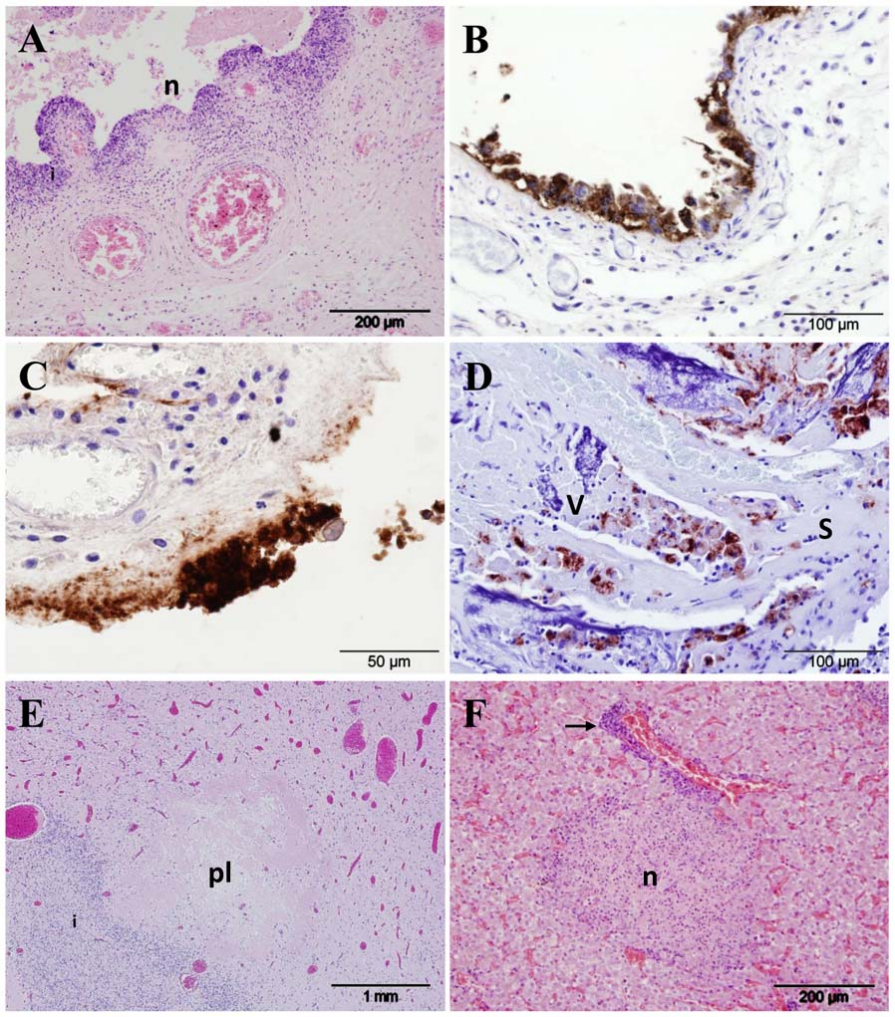
Histopathological changes and immunohistochemical detection of chlamydial antigen in sheep infected with *C. abortus*. (A) Cross-section of an affected placental membrane at 138 days gestation (dg) from a ewe intranasally administered 5×10^3^ IFU *C. abortus* (group 1), showing necrosis and sloughing of the chorionic epithelium (n) and inflammation in the underlying mesenchyme (i), HE. (B) Immunohistochemical labelling of *C. abortus* LPS in the placenta of a control ewe (group 5; subcutaneously infected with 2×10^6^ IFU *C. abortus*) at 141 dg; note intense labelling (brown colouration) of chlamydial inclusions and antigen in chorionic epithelial cells displaying signs of acute degeneration. (C) Immunohistochemical labelling of placenta sampled at 102 dg from a ewe intranasally administered 5×10^3^ IFU *C. abortus* (group 1), showing an area of diffuse and granular positive labelling for *C. abortus* antigen associated with the destruction and loss of the chorionic epithelium. (D) Cross-section through a placentome at 138 dg from a ewe intranasally administered 5×10^5^ IFU *C. abortus* (group 2), showing the interdigitating caruncular septum (s) and fetal placental villus (v). Note the light inflammation and pockets of positively labelled chlamydial antigen in the septal tissue and the proportionately greater villous labelling along with necrotic changes. (E) Section of fetal forebrain sampled at 138 days gestation from a ewe intranasally administered 5×10^3^ IFU *C. abortus* (group 1) showing periventricular leucomalacia (pl), HE. (F) Section of fetal liver sampled at 136 days gestation from a ewe intranasally administered 5×10^5^ IFU *C. abortus* (group 2), HE; note the focal necrosis (n) and periportal inflammation (arrow).

Placental tissues were available for examination from all but 4 ewes in the study. Lesions characteristic of *C. abortus* infection [7] were observed in the placental samples from groups 1, 2, 3 and 5 (Table 2). Histopathological changes consisted of suppurative placentitis with extensive disruption of the chorionic epithelium and associated aggregations of polymorphonuclear neutrophils (PMNs) along with arteritis (Figure 2A). Aborted/non-viable offspring were produced by 10 ewes in group 1 and the placentas of 7 contained lesions consistent with *C. abortus* infection and a further 3 had milder pathological changes (Table 2). In group 2 the placentas of 7 ewes that produced non-viable offspring had characteristic pathological changes, a further 2 had milder, less extensive changes and one was normal (placental samples were not retrieved from two ewes). In group 3, the placenta of the one ewe that aborted contained mild lesions, not necessarily specific for infection with *C. abortus*, whereas in group 5, all ewes that aborted had lesions characteristic of infection with *C. abortus* in the placenta. In all cases, when histopathological changes suggestive of *C. abortus* infection were observed, *C. abortus* DNA was detected by qPCR and organisms were also detected by mZN and by immunohistochemistry (IHC) using anti-LPS monoclonal antibody 13/5 (Figure 2B, C, D). Positive IHC labelling was observed to be in the form of intracytoplasmic inclusions in chorionic epithelial cells (Figure 2B). In placental samples with more advanced lesions, labelling was more diffuse and granular and associated with the destruction of the chorionic epithelium (Figure 2C).

In ewes that produced live viable lambs, from groups 1–3 and 5 (numbering 6, 6, 18 and 3, respectively), there were 2 placentas in group 1 with histopathological changes consistent with chlamydial infection, one in group 2 (mild), 3 in group 3 and one in group 5. In all cases, when characteristic lesions were observed a significant amount of *C. abortus* DNA was also detected by qPCR and organisms were also detected by IHC in intracytoplasmic inclusions or as more diffuse or granular staining, as for aborted animals. There were no histopathological changes found in the placentas from any ewes in group 4a, or from the 2 ewes in groups 3 and 4a that had lambs dying as a result of dystocia (Table 2). Also, no histopathological changes were detected to suggest infection by any other abortifacient agent. Similarly, in those ewes that aborted before 126 dg no bacteriological evidence was detected of other pathogens, such as *Campylobacter* and *Salmonella* spp.

In the event of abortion, stillbirth or death soon after birth the most consistent findings in lambs and foetuses were observed in the brains. Here lesions consisted of focal leucomalacia primarily located in the cerebral white matter, suggestive of anoxic damage (Figure 2E). Mild changes in the brain were observed in 5 lambs/foetuses from group 1, 6 from group 2, and one each from groups 3 and 5. In the liver, histopathological changes were also observed and consisted of semi-suppurative periportal hepatitis, and in one case from group 1 a focus of necrosis was also observed (Figure 2F). Mild histopathological changes in the liver were found in 9 lambs/foetuses from group 1, 12 from group 2, one from group 3 and 4 animals from group 5. Histopathological changes seen in the lung were mild and consisted of clusters of PMN in the alveoli, suppurative interstitial inflammation and in one case interlobular oedema and haemorrhage. These mild changes were found in 4 animals in group 2 and in one from group 3. In one instance, from an aborted foetus in group 5, there was a focus of PMN observed in the myocardium, otherwise no pathological changes were observed in the heart and there were no histopathological changes found in the tissues sampled from the dead lamb in group 4a.

### Serological Analysis

**Figure 3:**
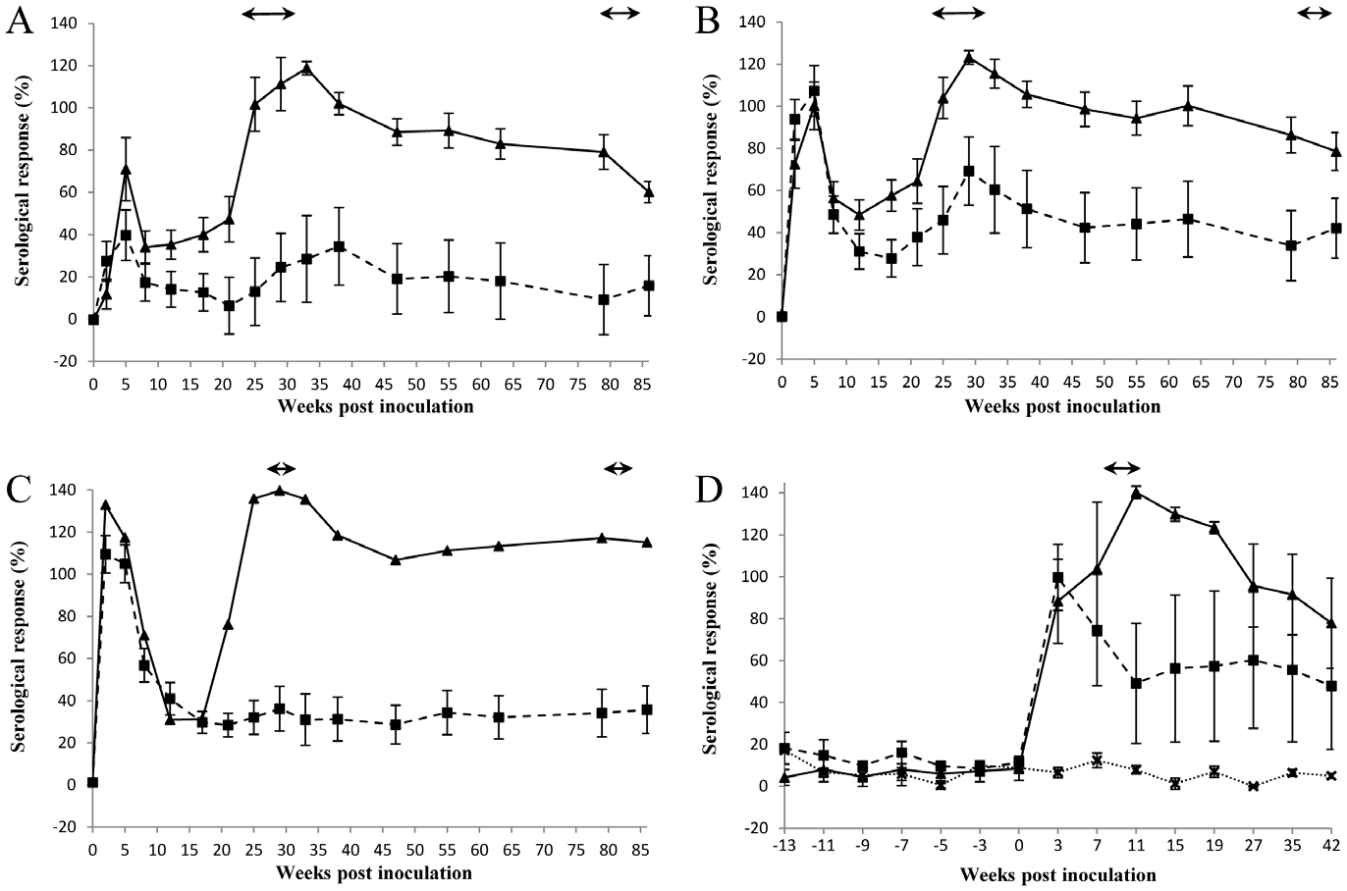
Serological responses in ewes that aborted (includes non-viable births and stillbirths) (-▴-) and lambed (-▪-) following intranasal inoculation with 5×10^3^ (A; Group 1), 5×10^5^ (B; Group 2) or 5×10^7^ (C; Group 3) IFU *C. abortus* or intranasally administered negative control inoculum (D; Group 4a; all lambed) (^…^x^…^) prior to pregnancy, or subcutaneously injected with 2×10^6^ IFU at 70 days of gestation (D; Group 5). Means (± SEM) of normalised responses (see Materials and Methods) are shown. 100% is equivalent to an OD_450nm_ of 2.25. The lambing/abortion periods for years 1 and 2 are indicated by the horizontal double-headed arrows. Year 1 only for Groups 4a and 5 (D).

Ewes were blood sampled prior to inoculation and at regular intervals throughout the course of the two lambing seasons. Following intranasal challenge, the highest and most rapid mean antibody responses were observed in group 3 animals, which received the highest dose of *Chlamydia* (Figure 3). All except 2 of the group 3 ewes (19 pregnant ewes; Table 1) were positive (>60%) at two weeks after inoculation. The mean antibody responses were lower and occurred slightly later in group 2 animals, where the challenge dose was less: 13 out of 18 ewes were positive 2 weeks after challenge. In group 1, where the lowest dose of *Chlamydia* was administered, only one of 16 animals had seroconverted two weeks after infection, which had only increased to 9 ewes by 5 weeks post infection.

The antibody profiles for all aborted ewes in groups 1 to 3 showed a similar trend. After an initial elevated response, following from the administration of the inoculum, the mean levels declined and then rapidly increased from around week 20 of pregnancy, with the mean response peaking at approximately week 30 coinciding with the majority of animals aborting. All aborted ewes in groups 1, 2, 3 and 5 were positive for *C. abortus* antibody at or immediately prior to the abortion, except for one ewe in group 1 that did not become positive until 4 weeks later.

Ewes that had infected placentas (with a high bacterial load) but produced clinically healthy lambs, also had elevated mean antibody titres at parturition. However, the mean levels were considerably lower than those observed for the aborted ewes and did not persist over such a long time period, and were mostly below the 60% ELISA cut-off for positivity. All ewes that produced viable offspring and had no clinical, microbiological, molecular or pathological evidence of infection were also negative for *C. abortus* antibody.

The interaction effect of ‘group’ and ‘lambing status’ was statistically non-significant (*P* = 0.161) indicating that the mean difference in antibody titres between groups did not differ for animals that either aborted or lambed normally. The interaction effect of ‘lambing status’ and ‘time’ was statistically significant (*P* = 0.017), with the mean antibody titres of animals that aborted being statistically significantly higher (*P_f_*<0.05) at all time points compared to those that lambed normally, irrespective of group. Similarly the rise in the mean antibody titre occurring in the lead up to abortion (weeks 13–26) was statistically significant (*P_f_*<0.001) for ewes that aborted, although the mean difference for the same period was not statistically significant (*P_f_* = 0.821) for ewes that lambed normally. Finally, the interaction effect of ‘group’ and ‘time’ was also statistically significant (*P* = 0.032), suggesting that the mean difference in antibody titres between groups varied across time post inoculation.

Blood sampling continued after lambing/abortion in year 1 and continued through to lambing in year 2 for groups 1–3. Results showed that, although *Chlamydia*-specific antibody levels of the aborted ewes steadily declined over the period of year 1, they continued to remain at an elevated level.

### Interferon-gamma Analyses

**Figure 4:**
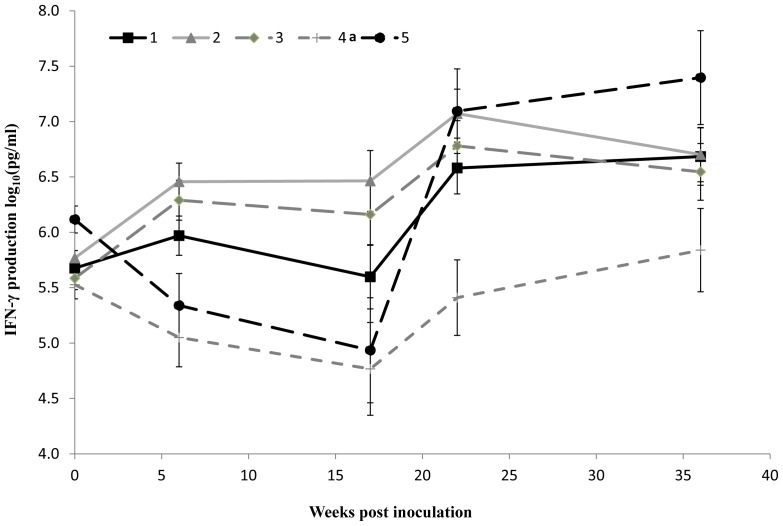
IFN-γ production from PBMC restimulated with UV-killed *C. abortus* antigen *in vitro*. PBMC was prepared from whole blood from ewes, restimulated *in vitro* with UV-killed *C. abortus* antigen and culture supernatants harvested for analysis using the BOVIGAM™ commercial ELISA to determine the concentration (in pg/ml) of IFN-γ produced (see Materials and Methods). The data are log_10_ transformed means (± SEM) of normalised responses for each group following intranasal inoculation with 5×10^3^ (Group 1 -▪-), 5×10^5^ (Group 2 -▴-) or 5×10^7^ (Group 3 -♦-) IFU *C. abortus* or negative control inoculum (Group 4a -+-) prior to pregnancy; or subcutaneous injection with 2×10^6^ IFU *C. abortus* at 70 days of gestation (Group 5 -•-).

Blood samples were taken from the ewes for IFN-γ analysis prior to inoculation and then at specific time points during the course of the first lambing season (at 6 and 17 weeks post inoculation (wpi), at the start of lambing/abortion (22 wpi) and 7 weeks following the lambing/abortion period (36 wpi) (Figure 4). Overall statistical analysis of data from groups 1, 2 and 3 showed no evidence of an effect of ‘group’ or ‘lambing status’ on the mean IFN-γ levels produced by PBMC stimulated with *C. abortus* antigen, indicating that the difference in mean IFN-γ production between animals that aborted or lambed normally was statistically non-significant, irrespective of the group.

Additionally, the mean levels of IFN-γ production by PBMC for groups 1, 2 and 3 at different time points were also compared with the negative control group (group 4a). Six weeks following intranasal inoculation with *C. abortus*, groups 2 (medium dose) and 3 (high dose) exhibited statistically significant recall responses to EBs as measured by the elevated mean production of IFN-γ by PBMC, when compared to the uninfected sheep (group 2 vs group 4a: *P_f_* = 0.004; group 3 vs group 4a: *P_f_* = 0.010), in contrast to group 1 (low dose), where no statistically significant difference was observed (*P_f_* = 0.057). These data should be viewed in the context that the values for groups 4a and 5 at week 6 were lower than they were at baseline. One month prior to the start of the abortion/lambing period (week 17), a clear effect of infection on the mean IFN-γ production by PBMC from the 2 groups infected with the highest doses of *C. abortus* compared to the uninfected groups (4a and 5) was observed (Figure 4). The mean IFN-γ production, relative to group 4a, was statistically significantly higher in groups 2 (*P_f_* = 0.001) and 3 (*P_f_* = 0.004), while the mean difference between groups 1 and 4a was again statistically non-significant (*P_f_* = 0.074). During this time, the mean IFN-γ level for group 5 was similar to that of group 4a. By the start of the lambing/abortion period at week 22, and following the subcutaneous infection of group 5 control animals at week 18, all four infected groups showed statistically significantly elevated mean levels of IFN-γ compared to the uninfected group 4a sheep (group 1, *P_f_* = 0.014; group 2, *P_f_* = 0.001; group 3, *P_f_* = 0.004; and group 5, *P_f_* = 0.001). By the final sample point (week 36) the only statistically significant mean difference in IFN-γ production was between groups 5 and 4a (*P_f_* = 0.002).

### Clinical Outcome and Pathogen Detection in year 2

The clinical outcomes in year 2 for each of the ewes in groups 1–4 are summarised in Table 3. All ewes lambed at normal gestation (146 days ±4 days), other than for one ewe in each of groups 2 and 3 that proved not to be pregnant. Four of the lambs were found not to be viable at birth and a fifth was euthanized within 48h of lambing on humane grounds. Placentas were recovered from all but 3 ewes and all appeared normal upon macroscopic examination. *C. abortus* organisms were not detected in any of the placentas or vaginal swabs taken from the ewes by either qPCR or mZN. Examination of histopathology samples supported the finding that there was no evidence of *C. abortus* infection. In the cases where lambs died, there was no microbiological, molecular or pathological evidence to suggest that any lamb deaths were associated with chlamydial infection.

**Table 3:** Year 2 clinical outcomes of lambing.

Group	Number of ewes	Number not in lamb	Number of singles/twins	Number of live/dead lambs	Number of placentas retrieved
1	16	0	12/4	18/2	14/16
2	17	1	5/11	26/1^a^	16/16
3	17	1	8/8	23/1	15/16
4b	10	0	8/2	11/1	10/10

a euthanised lamb.

## Discussion

Following experimental inoculation with *C. abortus*, ewes were observed to develop a short-lived rise in rectal temperature (febrile response), as observed in other studies [5], [8], [9], [11], [15], [31]. However, ewes given the largest dose of organisms (group 3) produced an earlier reaction, more animals were affected, and the febrile response was of a longer duration than in the other groups, with those in group 1 responding the latest with the least overall response. These results show a clear and statistically significant dose effect where the timing and magnitude of the febrile response is directly proportional to the amount of *C. abortus* administered.

Although the challenge control animals (group 5) showed a febrile response similar to that of group 3, they were most similar in terms of clinical outcome to those receiving the lowest doses (groups 1 and 2). Not only were they similar in terms of foetal mortality (abortion rates of 59, 67 and 50%, respectively for groups 1, 2 and 5), they were also similar in terms of timing, with abortions occurring an average 2–3 weeks prior to expected parturition. This timing is similar to that observed in natural field situations, where during an abortion storm losses affecting 30% or more of animals are reported [1], [32], as well as similar to that reported in other experimental studies [15], [33]. Although there was no overall statistically significant difference in foetal outcome between these groups the higher abortion rate in group 2 resulted from the greater number of non-viable lambs born in this group.

In contrast to groups 1, 2 and 5, foetal mortality was significantly lower in group 3, where only one ewe aborted at 115 dg, suggesting that animals in this group were protected in some way from infection. Indeed, the animals in this group had a normal mean gestational length of 141 days, very similar to that of negative control Group 4a (144 dg). However, it is clear from the bacteriological analysis, pathology results and quantified bacterial loads that 3 of the remaining 18 animals in group 3 that lambed normally had placental lesions and organism loads typical of EAE [15], [20]. Indeed, the number of bacteria detected in these 3 ewes (0.3–3.9×10^6^ organisms) was greater than in the ewe that aborted (0.2×10^6^ organisms) and contrasts with the numbers observed for the other 15 ewes that lambed (14–578 organisms). This suggests that there is a fine balance between organism load, pathological damage and pregnancy outcome. Regardless of this, the low organism load in the ‘normal’ lambed ewes still supports the view that this group of animals was protected to a much greater extent than in the other groups.

Despite the differences in pregnancy outcome, no pathological differences were observed in placental samples from aborted ewes from any of the groups, including our standard challenge group (group 5), and they were indistinguishable from those observed in previous experimental studies or in a natural outbreak [5], [7], [9], [11], [23], [25], [34]. Foetal samples showed evidence of infection in the liver, lung and to a lesser extent in the heart, and focal leucomalacia, suggesting hypoxic damage, secondary to the extensive pathological changes in the placenta, was observed in foetal brains, as previously described [7]. Additionally there was no statistically significant difference in mean antibody responses generated between animals that aborted, or between those that lambed, irrespective of group; although there was a clear significant difference at the time of abortion/lambing between those that aborted and those that lambed normally. Furthermore, there was an increased mean antibody titre observed in ewes that lambed normally in groups 1 and 2, but not in group 3, suggesting that despite the lack of bacteriological and pathological evidence a degree of infection and seroconversion took place in these animals.

As we and others have noted, once an animal has aborted or delivered a stillborn or weakly lamb they are generally considered to be immune and do not abort again as a result of EAE [1], [15], [20], [32], [35]–[37]. Indeed, this was the case in this study where all animals lambed normally at parturition in the second year, with no evidence of infection following bacteriological, molecular, serological or immunological analyses. Protective immunity is thought to develop at the time of parturition or abortion following a primary infection and is associated with seroconversion that occurs as a direct result of the massive multiplication in the number of organisms in the placenta in the lead up to expulsion [1].

Interestingly, the serological responses across all the groups immediately following inoculation resulted in a pattern that closely mirrored the febrile response. It is intriguing to note that the response was greatest and occurred faster in the group 3 animals that received the largest dose and in which the majority of ewes appeared to have developed a degree of immunity. As the dose of 5×10^7^ IFU induced less foetal mortality than the lower doses, it suggests that latent infection was not established to the same degree. It is not known whether this was due to more effective clearance of *C. abortus* or because a latent infection was established but kept in check by the immune system. As these ewes did not show any evidence of chlamydial infection in year 2 it is tempting to suggest that the higher dose provoked an immune response that cleared infection early on to a greater extent than in groups 1 and 2. Similar conclusions were drawn from early research conducted by McEwen et al. [6] who noted that the infectivity and pathological effects of a small dose was equal to or greater than the effects of a large dose of *C. abortus* when administered prior to mating. Whether such an immune response is cell mediated or antibody based, or a combination of both, is not clear, although protection studies against primary chlamydial infection has shown cellular responses to have a greater role than antibody, with IFN-γ regarded as a major immunological correlate of protection [27].

However, in this study, although the IFN-γ data indicated that the sheep mounted a cellular immune response to *C. abortus* following the intranasal infection prior to pregnancy, analysis of the data showed no statistically significant correlation between PBMC IFN-γ production and foetal outcome. This is despite the fact that infected sheep could be distinguished from non-infected sheep on the basis of recall responses, with the strongest recall responses observed in the infected groups at the time of abortion/lambing from week 22 onwards post inoculation, which is likely a reflection of chlamydial multiplication in the placenta at that time.

### Conclusions

Intranasal administration of *C. abortus* to non-pregnant ewes led, in the subsequent pregnancy, to infection of the placenta and to abortion. This route of administration therefore comes very close to reproducing the natural infection that results from sheep coming into contact with infected placentae and/or foetuses. As a working hypothesis it is suggested that following intranasal administration, chlamydial organisms adhere to the pharyngeal mucosa and are taken up by the NALT, and possibly also by the tonsils, to initiate infection. The outcome of infection was dependent upon the dose given, with lower doses manifesting in higher abortion rates than a high dose. We suggest that with a lower uptake of *C. abortus*, as seen in groups 1 and 2, infection was established but with insufficient immunological stimulation to induce protective immunity, permitting a latent intracellular infection to persist, possibly in lymphoid tissue [25]. It is known that recrudescence of a latent infection may occur in a subsequent pregnancy when, following chlamydial multiplication in the ewe, the bacteria enter the blood and the resultant chlamydaemia transports the infection to the placenta, which is particularly vulnerable to *C. abortus* after 90 dg [5]. Invasion of the placenta and then the foetus occurs [5], [23], [25] with the severity of the pathological process being influenced by the timing and the degree of chlamydaemia. In turn these are influenced by the effectiveness of the maternal protective immune response to *C. abortus*, which is dictated by the extent of the ewe’s initial exposure to the bacterium. Moreover, while a lower dose successfully established infection but was insufficient to stimulate protective immunity, a high dose appeared to stimulate a degree of protection. Results suggest that a higher uptake of chlamydiae, as indicated by a greater febrile response, was triggered in group 3 that in turn invoked a response that prevented the establishment of a latent infection but without seroconversion being detected. Infection induced in groups 1 and 2 appears to have been below the threshold required to stimulate protection, permitting a persistent, latent infection that could recrudesce during pregnancy. Understanding the subtleties of the mechanisms that underlie these observations is key to understanding *C. abortus* latent infections in sheep. Importantly, these findings will help in understanding the mechanisms of infection underlying latency and onset of disease, as well as mechanisms for stimulating protective immunity, which will aid in the development of novel therapeutics and vaccines for controlling infection.

## Supporting Information

Figure S1 **Survival curve showing the total proportion of animals in a particular group exhibiting a normal (<40°C) rectal temperature as time progresses following intranasal inoculation with 5**×**10^3^ (Group 1), 5**×**10^5^ (Group 2) or 5**×**10^7^ (Group 3) IFU** ***C. abortus*** **.**
(PPT)
