# Supplementary figures and images for: Intranasal Infection with Chlamydia abortus Induces Dose-Dependent Latency and Abortion in Sheep
Source: PLoS One. 2013 Feb 28;8(2):e57950. doi: 10.1371/journal.pone.0057950 (PMC3585262; doi:10.1371/journal.pone.0057950)

## Slide 1
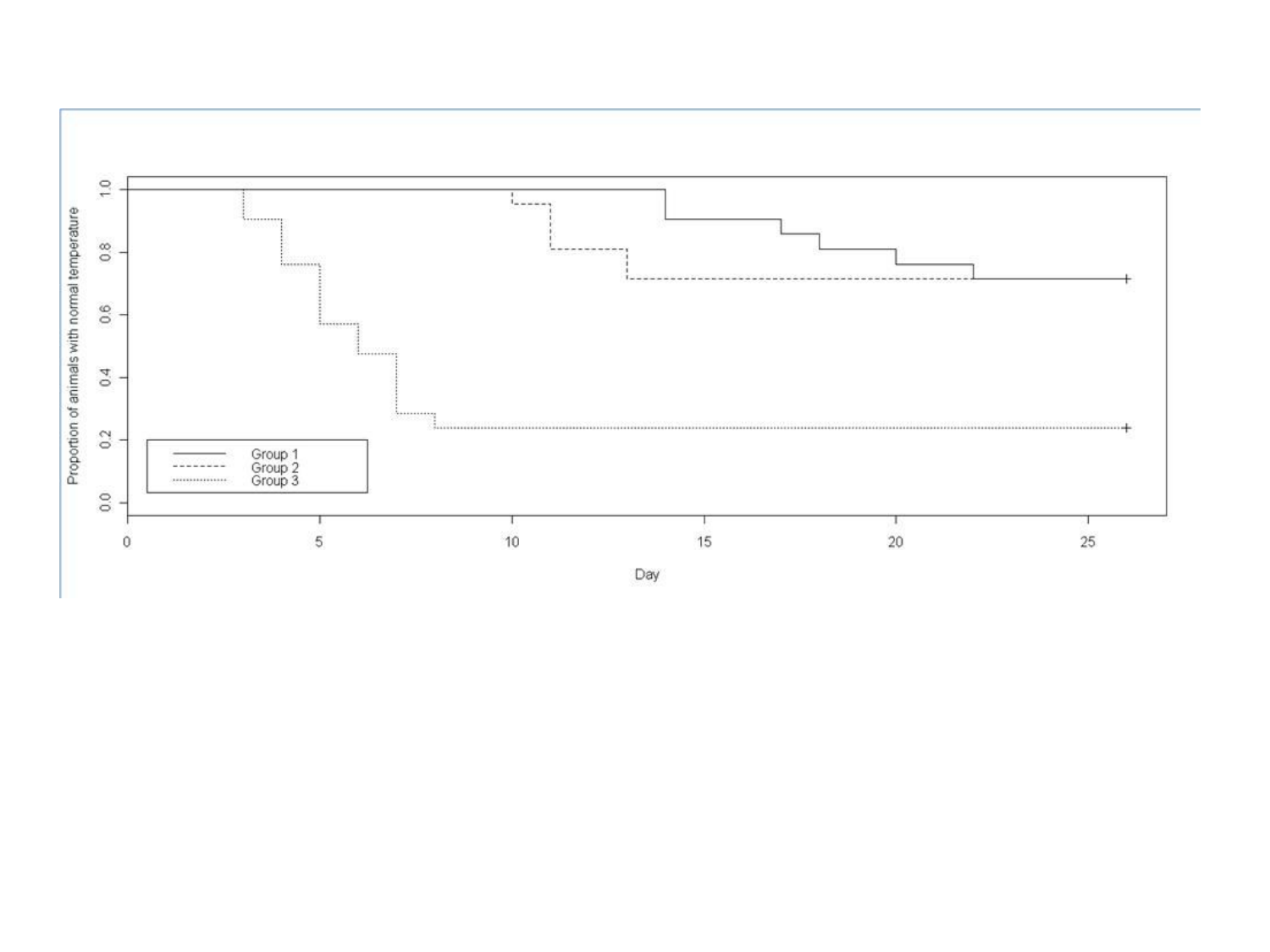

Supplement: Figure S1 — Survival curve showing the total proportion of animals in a particular group exhibiting a normal (<40°C) rectal temperature as time progresses following intranasal inoculation with 5×10^3^ (Group 1), 5×10^5^ (Group 2) or 5×10^7^ (Group 3) IFU C. abortus . (PPT) [file pone.0057950.s001.ppt]
